# Development of SiC–TiO_2_-Graphene neem extracted antimicrobial nano membrane for enhancement of multiphysical properties and future prospect in dental implant applications

**DOI:** 10.1016/j.heliyon.2022.e10603

**Published:** 2022-09-14

**Authors:** Mohammad Asaduzzaman Chowdhury, Nayem Hossain, Md. Abdus Shahid, Md. Jonaidul Alam, Sheikh Monir Hossain, Md. Ilias Uddin, Md. Masud Rana

**Affiliations:** a Department of Mechanical Engineering, Dhaka University of Engineering and Technology (DUET), Gazipur, Gazipur, 1707, Bangladesh; b Department of Mechanical Engineering, IUBAT-International University of Business Agriculture and Technology, Bangladesh; c Department of Textile Engineering, Dhaka University of Engineering and Technology, Gazipur, Gazipur, 1707, Bangladesh

**Keywords:** Nano membrane, Neem, PVA, Antimicrobial activity, Cytotoxicity

## Abstract

This paper presents the coating technology on Nano membrane using SiC–TiO2-Graphene with varying percentages of *Azadirachta indica* (Neem) extract with an objective to develop new coating materials. The nanomembranes have been synthesized by electrospinning machine over aluminum foil paper using the raw materials PVA grain, SiC, TiO2, Graphene, and neem. The nanomembranes have been characterized by SEM, XRD, FTIR, Surface Roughness, antibacterial, and Cytotoxicity test. FTIR analysis established the presence of PVA and neem indicating the formation of different organic compounds. It also confirmed that no chemical reaction occurred during the synthesis process. The membrane's roughness analysis obtained average roughness values from 1.15 to 3.84. The formation of homogeneous and smooth membranes with the formation of micropores was confirmed by SEM analysis. Miller Indices identified different types of crystal structures in XRD analysis. Antibacterial activity increased with the increase of the percentage of neem confirmed by the antibacterial test. No toxic effects were observed from the membrane during the cytotoxicity test. The obtained data confirmed that the synthesized nanomembrane could be used in different biomedical applications.

## Highlights

• Synthesis of Nano membrane using SiC–TiO2-GRAPHENE with varying percentages of *Azadirachta indica.*
• Synthesis by electrospinning machine.
• Increased antibacterial activity.
• Characterized by SEM, XRD, FTIR, Surface Roughness, Antibacterial and Cytotoxicity test.

## 1 Introduction

Nanotechnology is currently considered an effective tool to apply in material science research. Nanotechnology is used widely in a different range of applications, particularly in the field of energy, biotechnology, sensors, and related other areas. Nanotechnology has brought immense creativity and has pushed the limits of our understanding. The most recent technological advances will allow a much wider and more in-depth analysis of Nanoscale materials structures and functions. Nanotechnology and Material Science require integrating and comprehending the physical ideas shown by Biomaterials, nanometer-scale particle technology, and other nanotechnologies. Materials as well as innovative applications combining different areas of nanotechnology are invited. Scientists as well as engineers are brought together from different disciplines such as materials science, forensic engineering, biomedical engineering, chemical engineering, electrical engineering, physics, microtechnology, nanorobots and nanosensors [1, 2, 3]. Different researchers have developed different types of nanoparticles, namely Nanoparticles are categorized into various categories depending on their size, morphology, physical, and chemical properties. A few examples can be ceramic nanoparticles, semiconductor nanoparticles, lipid-based nanoparticles, carbon based nanoparticles, metal nanoparticles, and polymeric nanoparticles [4].

Nanomembrane can be defined as an organic, inorganic, or quasi-2D artificial freestanding or free-floating structure having a thickness less than 100 nm [5]. In the field of the air filter membrane, nanomembrane synthesized by electrospinning technique is a vast field for research in the current era [6, 7, 8, 9]. Low-pressure drops but high filtration efficiency can be achieved because of the low diameter, high surface-to-volume ratio, and high porosity which enables the electro-spun nanomembrane to intercept the fine particles [10, 11, 12]. The desired shape can be obtained by cutting nanomembranes to match tissue defects. Incorporating bioactive agents and nanoparticles onto the surface of nanomembrane are easy during the electrospinning technique to achieve desired properties [13].

The electrospun nanomembranes are being developed by incorporating different bioactive nanoparticles to improve tissue regeneration ability. Different nanoparticles such as bioactive glass nanoparticles [14], graphene oxide [15], nano-hydroxyapatite [16], cerium oxide nanoparticles [17], and gold nanoparticles [18] have been incorporated with electrospun nanomembranes to make nanomembrane more useable in biomedical applications. Maximum absorption coefficients with lower frequencies and larger cavity distance were obtained by incorporating PVA with nanomembrane [19]. Carbon nanotube and graphene are extensively incorporated with nanomembrane during the electro-spinning process because of their excellent mechanical, thermal, and chemical properties [20]. Graphene as a nanofiller incorporated with nanomembrane improves the biocompatibility and mechanical strength of the nanomembrane composite considerably [21]. Different nanofillers should be addressed and incorporated with electrospun nanomembrane to improve their properties and find more usability.

Titanium dioxide nanoparticles have a wide range of applications such as reducing toxicity in dyes and prescription drugs; wastewater treatments; silkworm reproduction; space applications; food industry; etc. For industries, the atmosphere, and agriculture, the applications of nanoparticles synthesized with a biological approach would be favorable [22]. In order to avoid the complexity of the formation of SiC Nano-particle by using graphene sheets during the ball-milling process, a revolutionary technique was used [23]. SiCnp/7075Al composite carbide nanoparticle-reinforced 7075Al (SiCnp/7075Al) has been made to 7075Al alloy after the therapy of T6 by a sequence of polymers, shift-speed ball milling, hot pressing and hot extrusion, and precipitation behavior [24].

The tribological behavior of graphite nanoparticles were evaluated using vegetable based oil as lubricant with the help of a pin on disc tribometer. Investigation was performed on graphite nanopartilces grease instrument. The results indicated that the vegetable based oil reduced the friction and wear significantly [25].

In this study, an attempt has been made to reduce the toxicity and bacterial effect of the multi-nanoparticle's membranes for biomedical applications by infusing nanoparticles with the nanomembrane. The toxic and bacterial impacts of SiC–TiO2- Graphite membranes are purified by 5%, 10%, and 15% of *Azadirachta indica* (Neem) extracts. In our real life, people suffer several kinds of bone diseases in which bone fracture very vital problem. So, in medical science, they try to reduce bone fracture by using metal. But metal is not safe for such a kind of operation. Metals contain toxic features and have bacterial effects. Therefore, this experiment will reduce those kinds of harmful effects of metals.

The novelty of this study is to use SiC–TiO_2_-Graphene hybrid nanoparticles in PVA solution to increase the versatile multifunctional aspect of this bio-based nanomembrane to be applied as a biomaterial in different biomedical applications which cannot be obtained when a single nanoparticle is used. In nanofibrous membrane, a large surface area to volume ratio, flexibility in surface functions, and increased stiffness and tensile strength when compared to other materials since fiber diameters are lowered from micro to nanoscale. There is large surface porosity with small pore size in nanomembrane when the nanoparticles are absent there. This type of porous nanomembrane is suitable for water filtration, high energy generation, and some other areas. But when nanoparticles are considered as nanofillers coatings in PVA nanomembrane, the porosities are disclosed and reduced to enhance the stiffness, strength, wear resistance, fatigue resistance, reduction of thermal expansion, and dimensional stability. These nanoparticles also have their inherent properties; these have also a considerable effect on nanomembrane properties. The concept of this type of nanofibrous-nanofiller membrane can be used in dental implants to mitigate the complication of stress shielding and implant-related infections and inadequate osseointegration problem.

**Figure 1:**
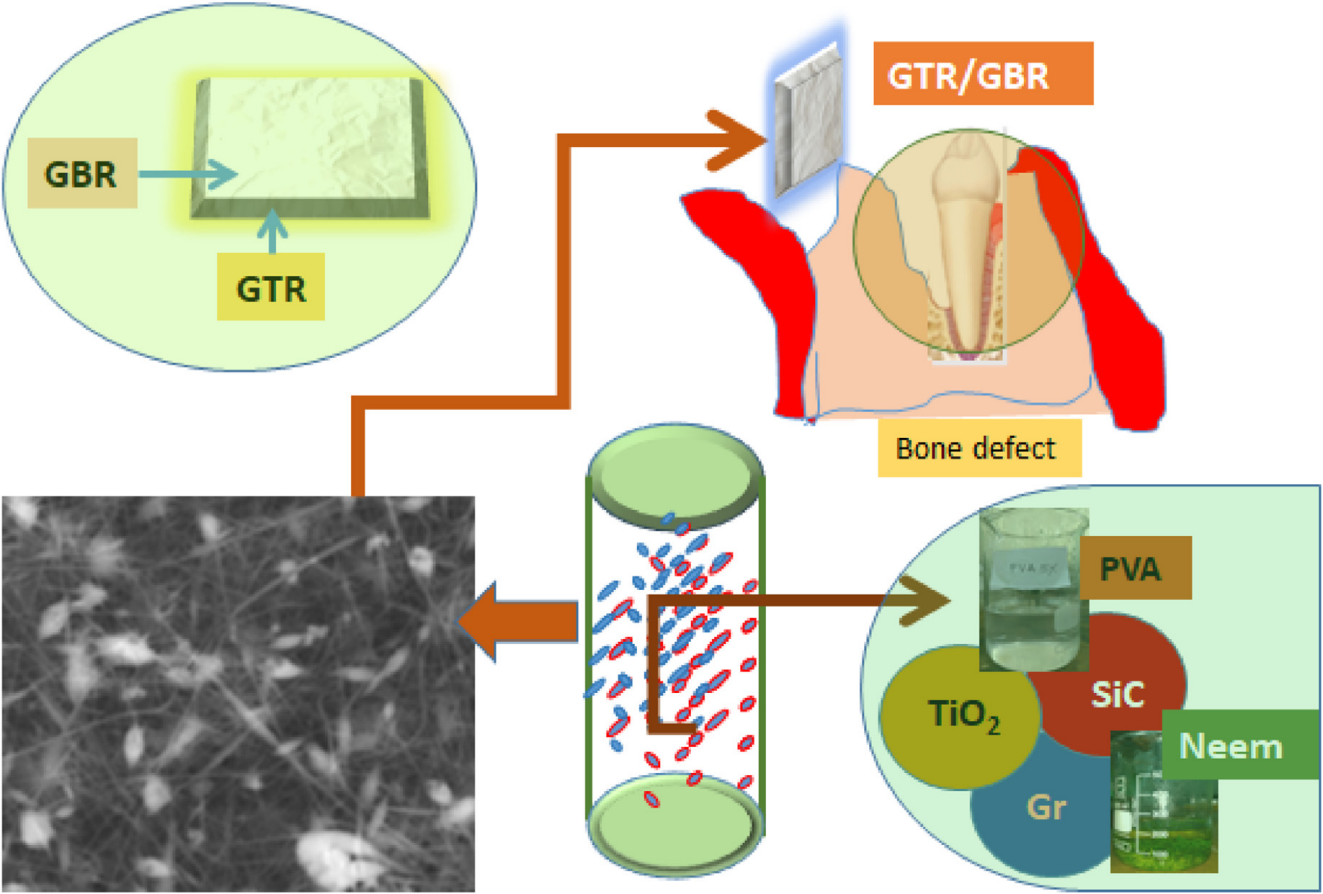
Concept of nanofibrous-nanofiller electrospun membrane in GTR/GBR dental application.

Besides this, GTR (guided tissue regeneration) and GBR (guided bone regeneration) are surgical methods used to regenerate the tooth-supporting tissues (GTR) and the alveolar bone (GBR) in edentulous areas, respectively (GBR). Recent techniques include the use of resorbable membranes/nanofiller. These materials have the advantage of disintegrating over time, eliminating the need for surgery to remove the scaffolds. Nanofibrous scaffolds have improved mechanical qualities, cell adhesion, biocompatibility, and antibacterial properties for GTR/GBR applications. This study can be used as a guideline for future research to resolve the problem using this type of nanofibrous-nanofiller membrane but before using this concept biodegradability should be ensured. Therefore, the choice of nanofiller in the nanofibrous membrane is important. The concept of the nanofibrous-nanofiller electrospun membrane in GTR/GBR dental application is illustrated in Figure 1.

Titanium and its alloys are recognized one of the best biomaterials for dental implant applications for their excellent properties such as higher tensile strength, higher corrosion resistance, and effects resistance due to body fluid, flexible and ferromagnetic abilities. Unique mix strength, biocompatibility, and low reactivity made them suitable for biomedical applications [26], as well as various surface modification and functionalization options [27]. Despite their many advantages, titanium-based materials in dental implants suffer. (i) Stress threshold (ii) Implant-related infections and insufficient osseointegration may inadequate the long-term viability of Ti-based dental implants, and (iii) corrosion issues [27, 28]. The graphene nanofillers are incorporated in this membrane since they increase osseointegration of implants, as well as graphene, which has good antimicrobial properties [29]. It is well known that bacterial infections are still the cause of dental implant failures. As a result, antibacterial titanium surface modification is critical. Although Ti-based materials have improved implant durability, there is still an opportunity for innovation to reduce titanium corrosion. Due to its higher strength, lower rate of reaction to the oral environment, and ease of deposition, silicon carbide (SiC) appears to be a viable material for protecting titanium from corrosion [30]. Make sure that the SiC film adheres to the patient's oral cavity and that the coating osseointegrates. The SiC inclusion must remain intact to maximize the benefits of this material during and after torquing the implant into the bone.

To ensure ion releasing, ion element identification, survival rate, non-toxicity, and cell growth confirmation, corrosion tests in artificial body fluids such as SBF, HBSS, in vitro test, cell viability test of degraded corroded solution using fluorescence microscopy, microwave plasma atomic emission spectroscopy, and cell attachment test using the chemical process are conducted. These tests are not observed in this study as there are many results available in the literature [31, 26, 27, 28, 29, 30]. Although, there is a limitation to doing these tests in our laboratory. Apart from this, this study is to understand the metaphysical properties of hybrid nanomembrane for promising application in dental implants in the future. But in the future, these tests will be carried out elaborately considering all aspects of nanomembrane-nanofiller coating integration with PVA and plant extract or other combinations.

## 2 Materials and methods

### 2.1 Materials

The materials used in this experiment are PVA, SiC nanopowder, TiO_2_ nanopowder, and *Graphene nanopowder*. PVA is collected from sigma, having a molecular weight of 98,000g (99% hydrolyzed). SiCnano powder, TiO_2_ nanopowder, and *Gr nanopowder* are purchased from Shanghai Ruizheng Chemical Technology Co., Ltd of China. Their chemical and physical properties are available in the literature [32, 33]. Neem leaves are collected from the university garden.

### 2.2 Preparation of plant extract

**Figure 2:**
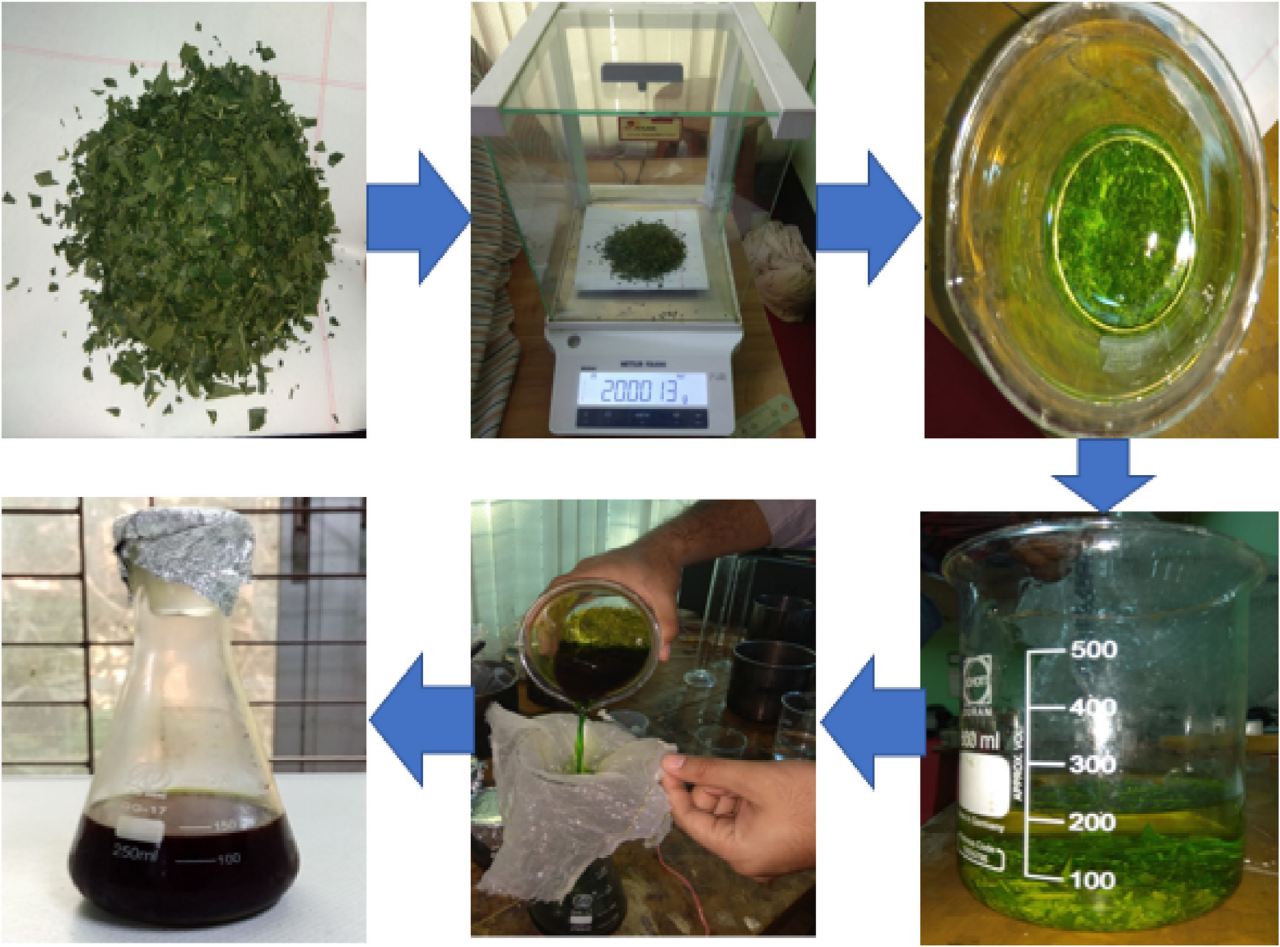
Extract preparation process of neem.

Neem leaves were collected from the Dhaka University of Engineering and Technology garden during the winter season. The tree was around three meters tall. The collected leaves are washed three times with distilled water to remove the dirt and other impurities and then dried under the sun. Then the leaves are cut into small pieces. 20 gm of leaves are taken and kept in a glass jar with 200 ml ethanol and kept at 4 °C for 24 h. The next day, the solution turned to greenish color. The solution is then filtrated to remove any solid particles and kept in the refrigerator for further use (Figure 2).

### 2.3 Preparation of common solvent PVA

15 gm of PVA is put in a beaker and mixed with 300 ml distilled water at 70 °C temperature over a magnetic stirrer to form a uniform solution.

### 2.4 Synthesis and development of nanomembrane

**Figure 3:**
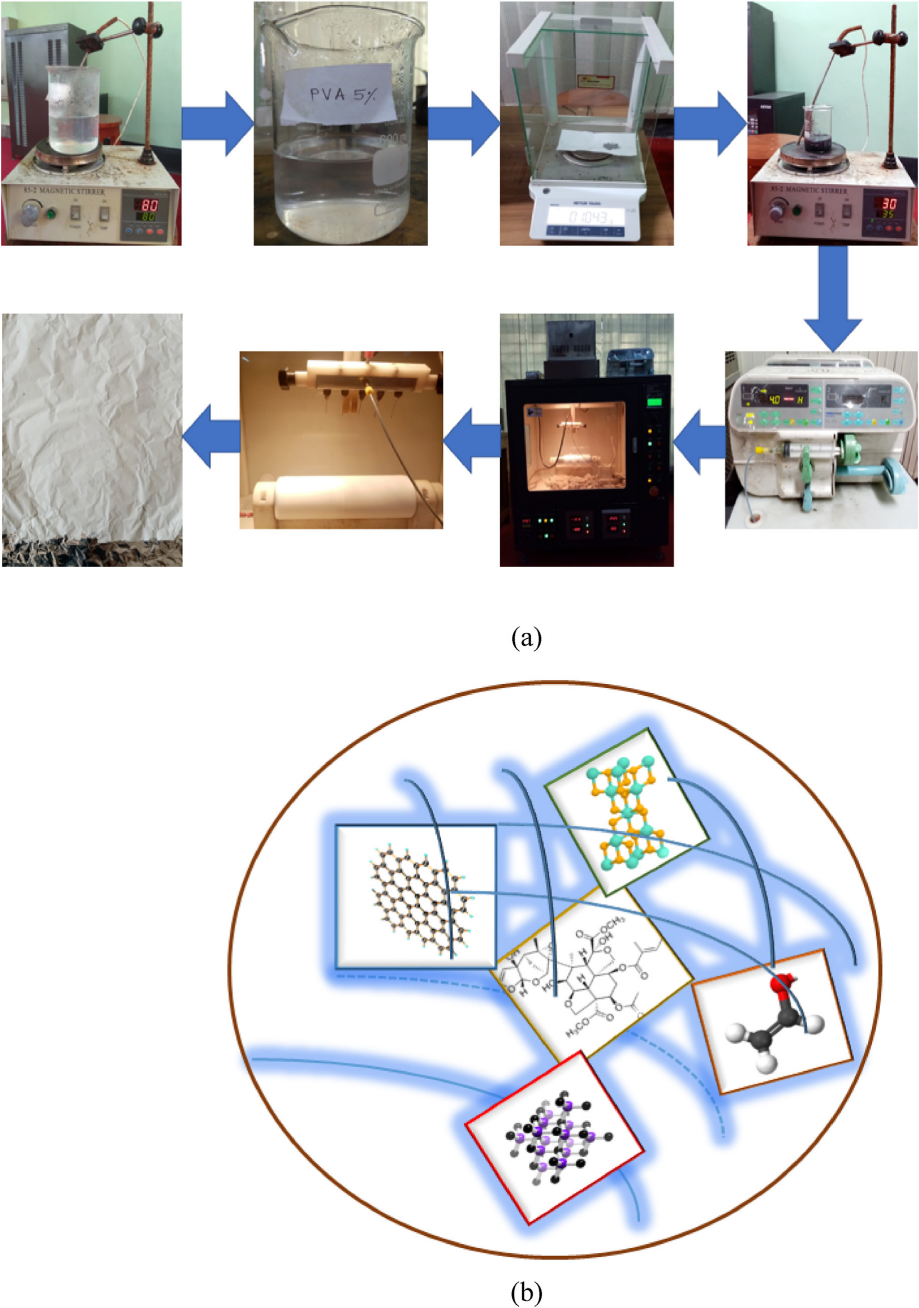
(a) Nanomembrane fabrication process, (b) Hybrid chemically bonded nanofibrous membrane.

0.2 gm SiC, 0.2 gm TiO_2_, and 0.2 gm *Graphene nanoparticles* are mixed together at room temperature by continuous stirring to obtain a combined mixture and the mixture was then dissolved with 20 ml PVA solution under a magnetic stirrer and kept in three different jars. The solution was stewed overnight at room temperature to remove bubbles. Then 0%, 5%, 10%, and 15% neem were added to the mixture in the jar. Later, the solution was injected into a syringe with having 0.9 mm inner diameter with a metal nozzle shown in Figure 3a. The nonwoven fabric of the drum collector was used to gather the nanofibers. The details mechanism of nanomembrane fabrication is as follows:

In the electro-spun process, the syringe is filled with the requisite solution. The syringe is inserted into the pump and joined by a pipe to some needles. The outside of the drum collector is wrapped in aluminum foil. The positive and negative voltage terminals are linked to the needles and the collector, respectively. In relation to SiC–TiO2-Gr and NEEM extraction on the surfaces of aluminum foil, a magnetic field is produced between needles and the collector as a result of positive and negative voltage potentials, which electrifies the liquid droplet of the solution to produce a jet. The jet is then stretched and extended to produce a nanofiber network.

The electrospinning had a 15 cm distance between the fiber collector and spinneret, 25 kV power supply, 0.5 mL/h spinning solution feed rate, and 25 °C temperature. Later, the membrane was dried at room temperature. Figure 3(b) shows a hybrid chemically bound nanofibrous membrane made up of PVA as a binder, nanoparticles as a reinforcing phase, and neem extract as a bioactive ingredient. PVA and neem extract react chemically to strongly embed nanoparticles in nanoscale fibers by adhesion process.

### 2.5 Characterization

#### 2.5.1 Fourier transforms infrared spectroscopy (FTIR)

FTIR analysis has been performed by FTIR spectrometer made by PerkinElmer, the USA maintaining the frequency range from 500 cm^−1^ to 4000 cm^−1^.

#### 2.5.2 Surface roughness properties

The surface roughness of the synthesized nanomembrane was estimated by a surface harshness analyzer (Taylor Hobson Surtronic S128, made in the USA). Three areas of the surface were estimated at any rate and the average value was considered.

#### 2.5.3 Scanning electron microscopy

The microstructure of the surface of the synthesized nanomembranes was examined by a scanning electron microscope made by Hitachi, Japan having model number SU1510.

#### 2.5.4 Surface topography

The SEM images were analyzed then by SPIP software for better realization of the microstructure of the surface by 2D and 3D images.

#### 2.5.5 Particle analysis

The SEM images were further analyzed by SPIP software as well to analyze the particle distribution on the surfaces of the synthesized nanomembrane.

#### 2.5.6 X-ray diffraction analysis

The synthesized nanomembranes were analyzed by Bruker D8 advanced X-ray beam diffraction analyzer which was made in Germany. The X-ray beam force was estimated in the scope of 100 ≤ 2θ ≤ 600 with a sweep speed of 0.50 min^−1^.

#### 2.5.7 Antibacterial assay

The antibacterial test of the nanomembranes was accomplished by applying the Kirby-Bauer disk diffusion test method maintaining the ASTM E2149-01 standard. The bacterial cell *Staphylococcus aureus* (ATTC 25923) as gram-positive bacteria were subjected to the tests in the disc having a size of 6 mm × 6 mm. The bacterial cell concentration was 1000 CFU/ml and the nanomembrane sample concentration was 200 mg/ml. The bacterial cells used in this research were collected from Dhaka University. The culture was spread on a nutrient agar plane after suspending in nutrient broth. The culture was incubated at 37 °C for 24 h. The culture was picked off from the agar plate with an inoculating loop and two single colonies were suspended in a nutrient broth that had a 10 mL volume. Later, the culture was incubated for 18 h at 100 rpm and 37 °C. 0.3 mM M and pH 7.2 sterile buffer solution were used to dilute and a final concentration of 1000 CFU/ml was obtained.

#### 2.5.8 Cytotoxicity test

The cytotoxicity tests of the nanomembrane were performed in a biological biosafety cabinet model NU400E, incubator, Trinocular microscope with camera, and Hemocytometer. All the equipment is made by Nuaire, USA. Vero cell line, a kidney epithelial cells extracted from an African green monkey, was maintained in DMEM (Dulbecco's Modified Eagles' medium) containing 1% penicillin-streptomycin (1:1) and 0.2% gentamycin and 10% fetal bovine serum (FBS). Cells (1.5 × 104/100 ul) were seeded onto a 48-well plate and incubated at 37 °C + 5 % CO2. The next day, a sample (Autoclaved) was added to each well. Cytotoxicity was examined under an inverted light microscope after 48h of incubation. Duplicate wells were used for each sample. Vero cell line, a kidney epithelial cells were supplied by the WAFFEN Laboratory, Dhaka, Bangladesh.

## 3 Results and discussion

### 3.1 Fourier transform infrared spectroscopy (FTIR) analysis

**Figure 4:**
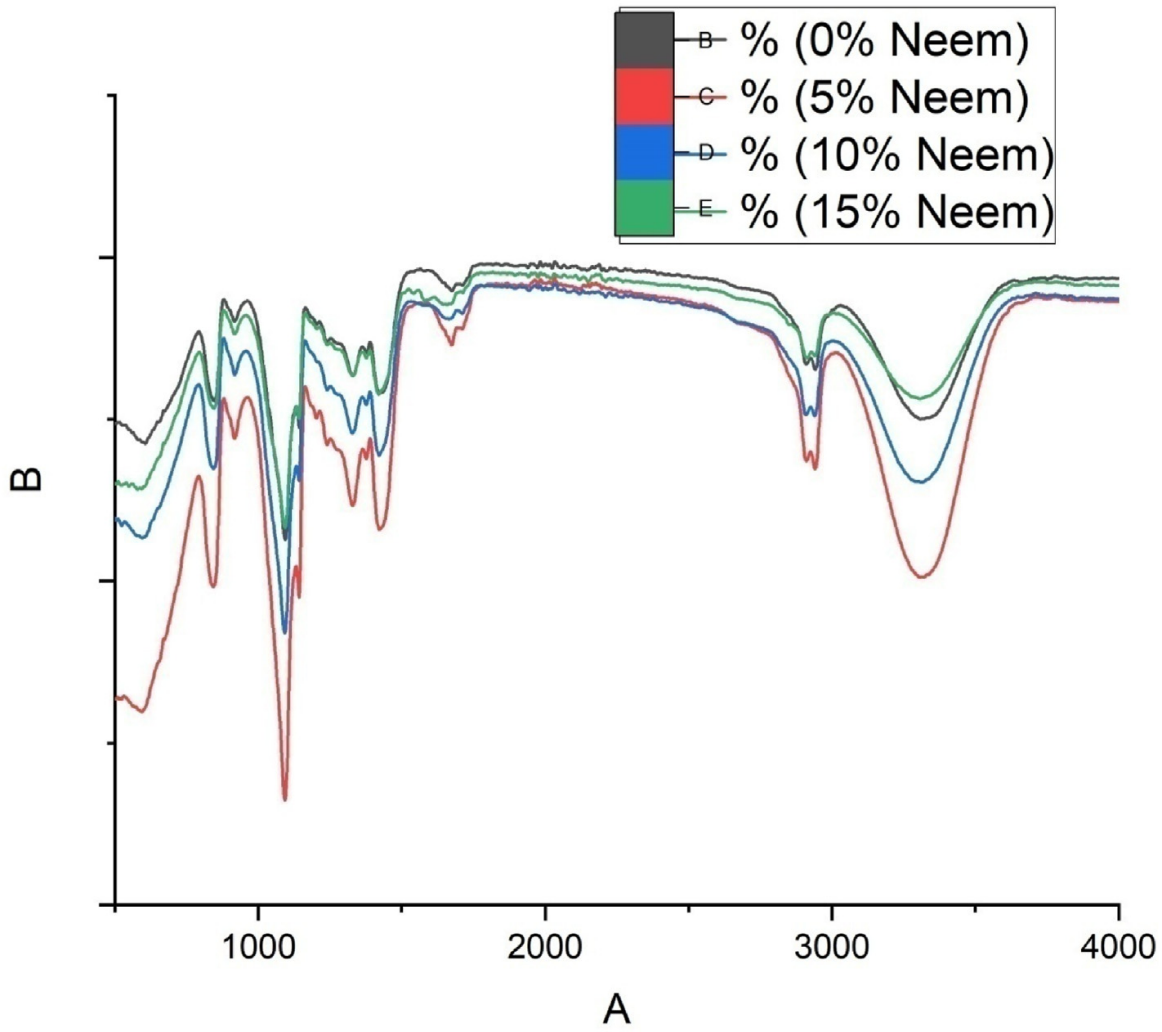
FTIR spectroscopy of nanomembrane samples.

Figure 4 shows the FTIR spectra of the developed nanomembrane samples and confirmed the presence of different functional groups in the synthesized nanomembranes. The graphs indicated that the presence of neem made the peaks sharper. Relatively stronger alkyl amine stretching vibration is attributed ​at 1092 cm^−1^ because of less oxidation effect due to the absence of neem. Alkyl amine stretching vibration is shifted to 1090 cm^−1^ in the nanomembrane containing 5% neem extract due to oxidation. Further shifting of alkyl amine stretching vibration is observed attributed to 1088 cm^−1^. Medium stretching functional group O–H is attributed to 3320 cm^−1^ [34]. Besides, medium stretching functional group alkane C–H is attributed to 2942 cm^−1^ [35]. Medium bending alkane is observed at 1422 cm^−1^ [36]. The presence of some other active functional groups is observed in the samples such as alkanes, ketones, carboxyl acid, amides, carbon monoxide, and alcohol. Based on the FTIR analysis, no chemical interaction occurred during the electrospinning process which maintained the original antibacterial properties of neem. Table 1 shows the presence of different active compounds in the developed nanomembrane.

**Table 1:** FTIR analysis data table of the synthesized nanomembrane.

Band (cm^−1^)	Functional class	Assignment	Vibration type
3320 cm^−1^	Amines and amides	N–H, C–H, O–H	Medium stretching
2942 cm^−1^	Alkane	C–H	Medium stretching
1674 cm^−1^	Alkene	C=C	Weak stretching
1422 cm^−1^	Alkane	C–O, C–H	Medium bending
1328 cm^−1^	Phenol	O–H	Medium bending
1092 cm^−1^	Alkyle amine	C–N	Strong stretching
594 cm^−1^	Halo compound	C–Br	Strong stretching

### 3.2 Surface roughness properties analysis

**Figure 5:**
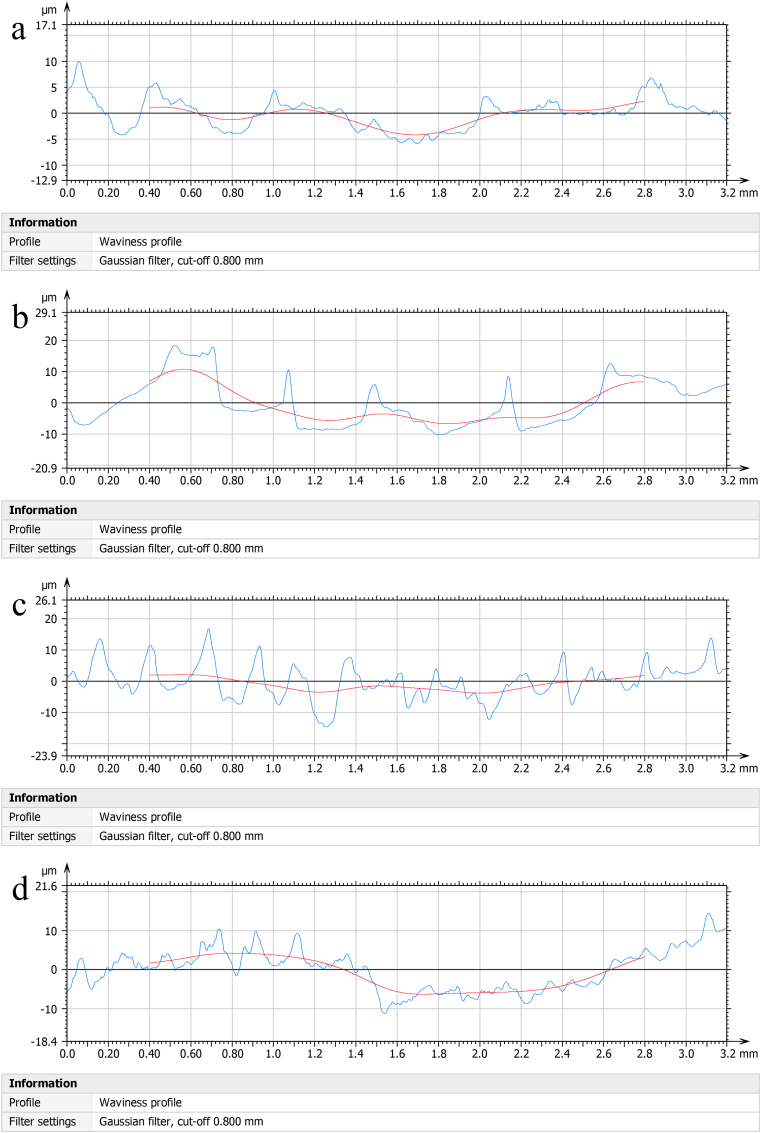
Roughness analysis of the samples at (a) 0% Neem, (b) 5% Neem, (c) 10% Neem, (d) 15% Neem.

**Figure 6:**
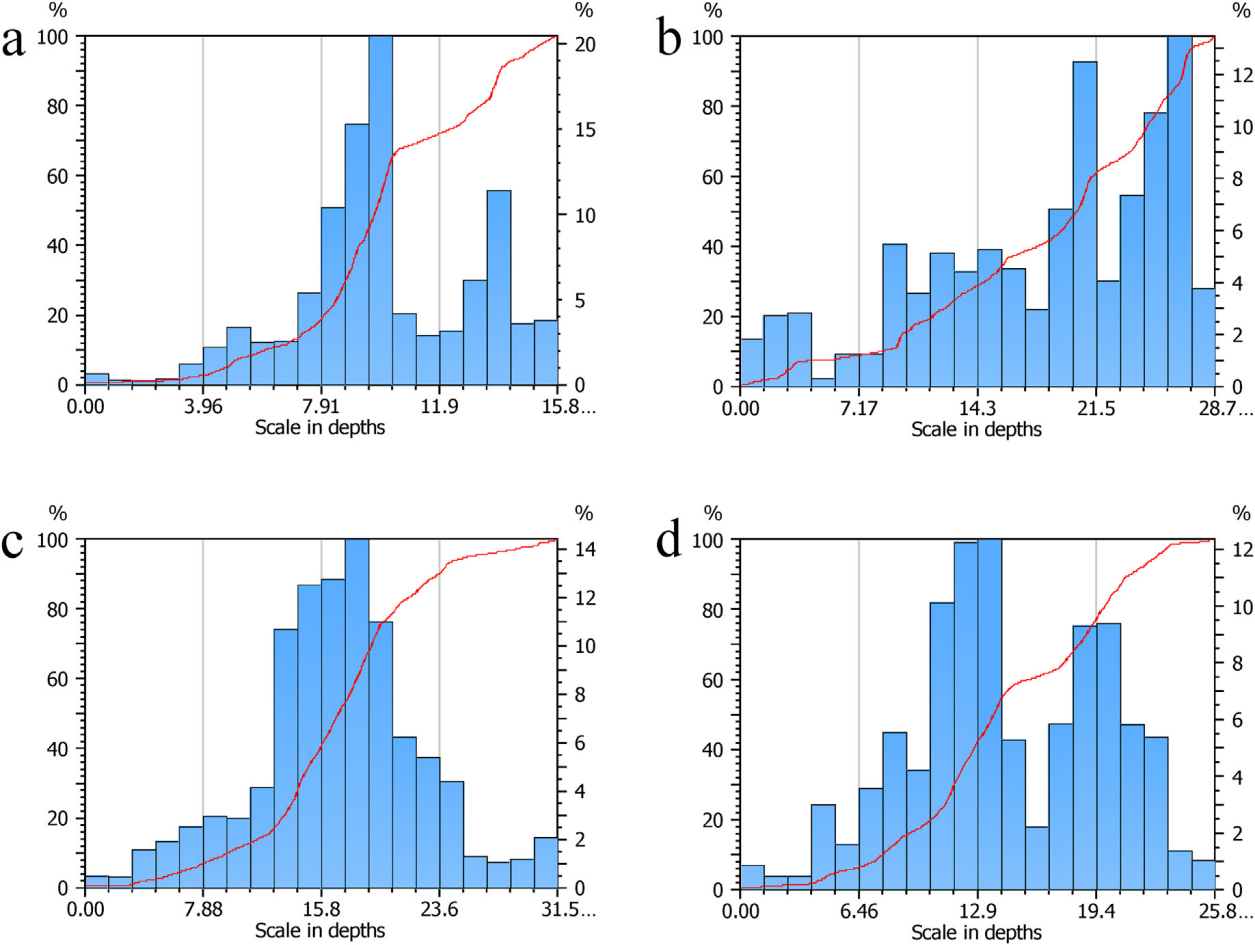
Roughness analysis of the samples with scale in depth at (a) 0% Neem, (b) 5% Neem, (c) 10% Neem, (d) 15% Neem.

Figures 5 and 6 shows the surface roughness analysis of the developed nanomembrane. Figures 5(a) and 6(a) shows average surface roughness Ra = 1.15μm, Figures 5(b) and 6(b) Ra = 3.32μm, Figures 5(c) and 6(c) Ra = 3.84μm and Figure 5 (d) and 6(d) Ra = 1.69μm. The results indicated that surface roughness increases with the increased percentage of neem due to surface irregularities up to a certain percentage. Depth concentration increases on the higher scale with the addition of neem shown in Figure 6. The highest depths in roughness occurred with 5% neem addition. More concentrated depths occurred when 10% neem is added. With a 15% addition of neem, the depths are distributed. Surface roughness is important to determine the osseointegration of dental implants. Moreover, bone-to-implant is increased by the rough surface [37]. Research has been carried out to find the relationship between surface roughness and bone cells' healing of the tissue surrounding dental implants [38]. Mechanical stability of the implant in host bone, adhesion of osteogenic cells, and entrapment of fiber protein are encouraged by the surface roughness. In vitro studies show that a positive correlation is observed between cellular attachment, cell proliferation, and surface roughness [39].

### 3.3 Scanning electron microscopy analysis

**Figure 7:**
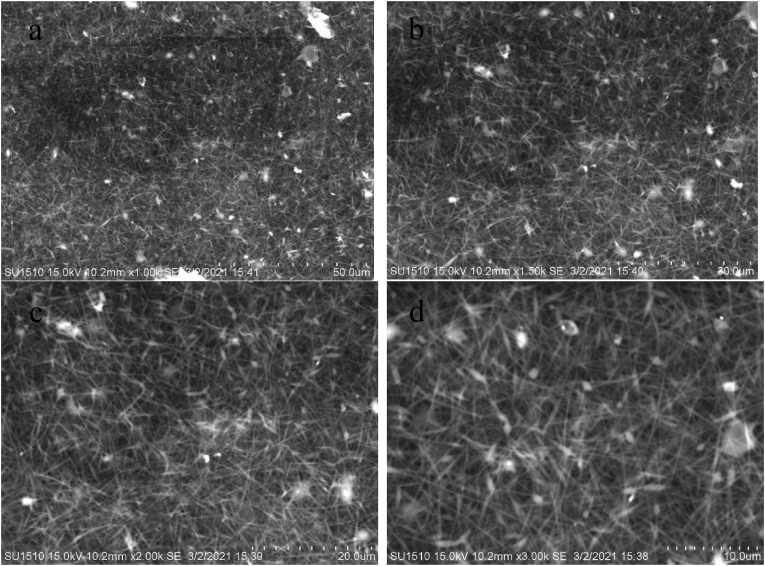
SEM analysis of the developed nanomembrane sample with 0% neem at different resolutions (a) 50 μm, (b) 30 μm, (c) 20 μm, and (d) 10 μm.

**Figure 8:**
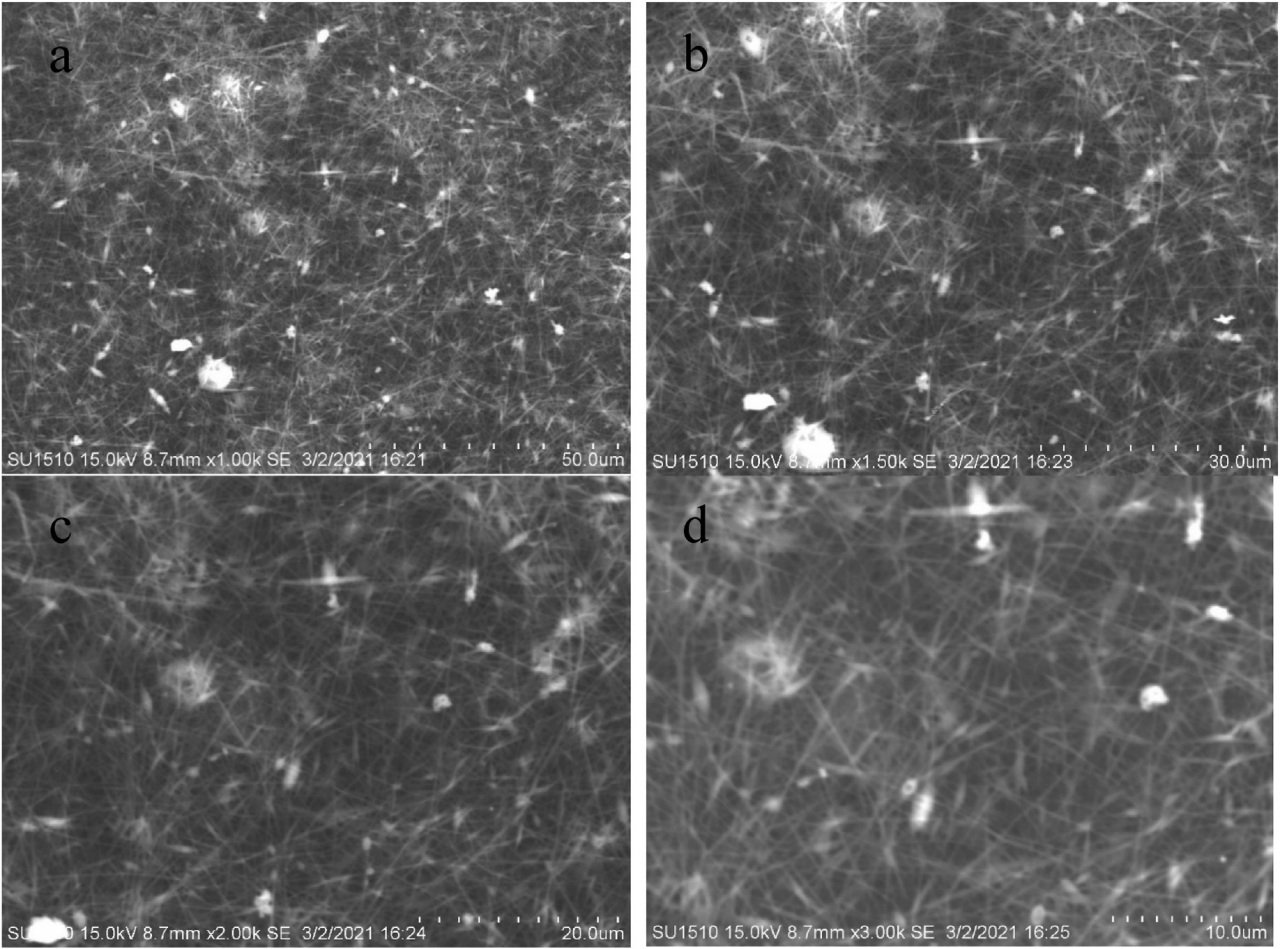
SEM analysis of the developed nanomembrane sample with 5% neem at different resolutions (a) 50 μm, (b) 30 μm, (c) 20 μm, and (d) 10 μm.

**Figure 9:**
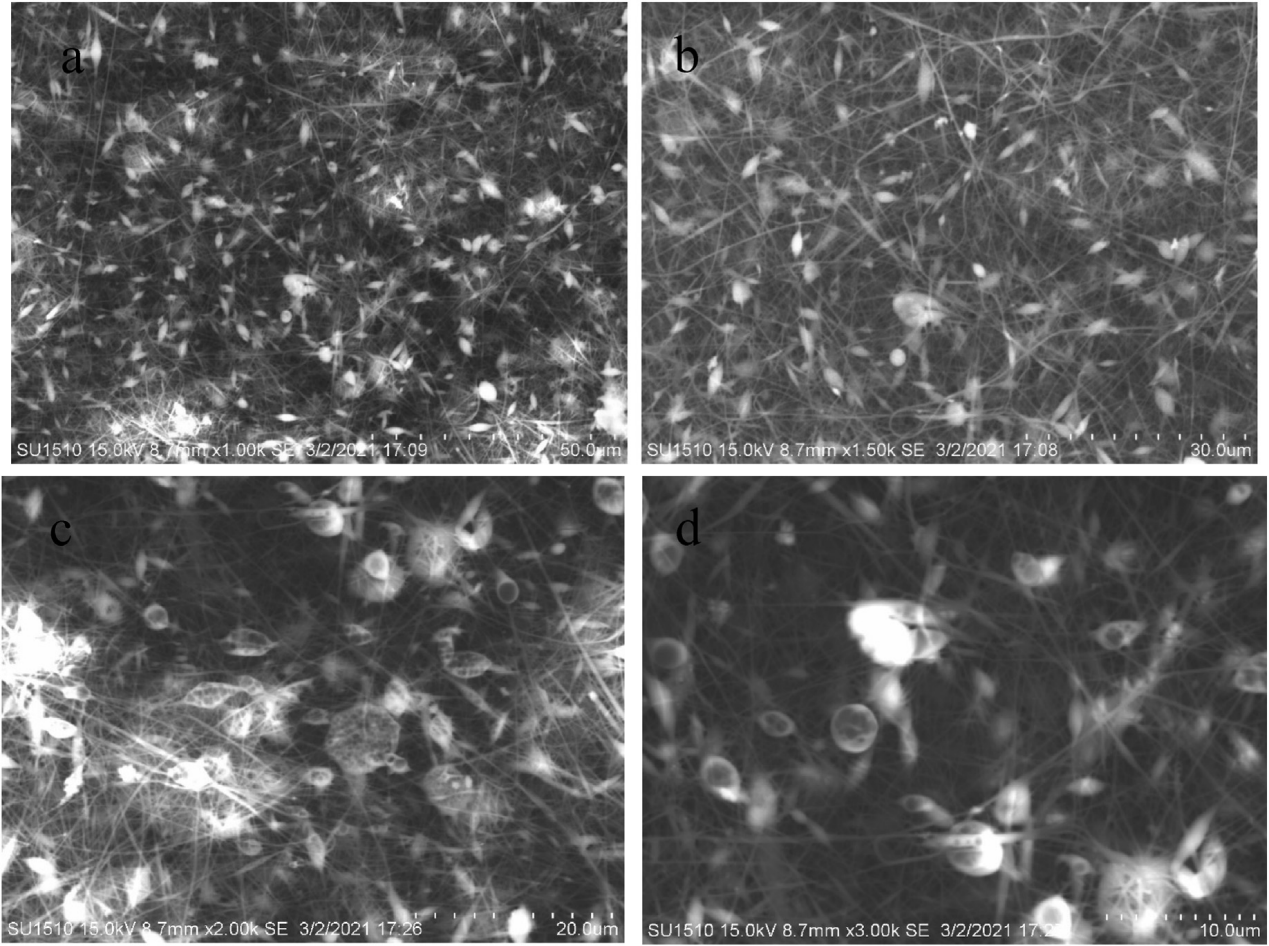
SEM analysis of the developed nanomembrane sample with 10% neem at different resolutions (a) 50 μm, (b) 30 μm, (c) 20 μm, and (d) 10 μm.

**Figure 10:**
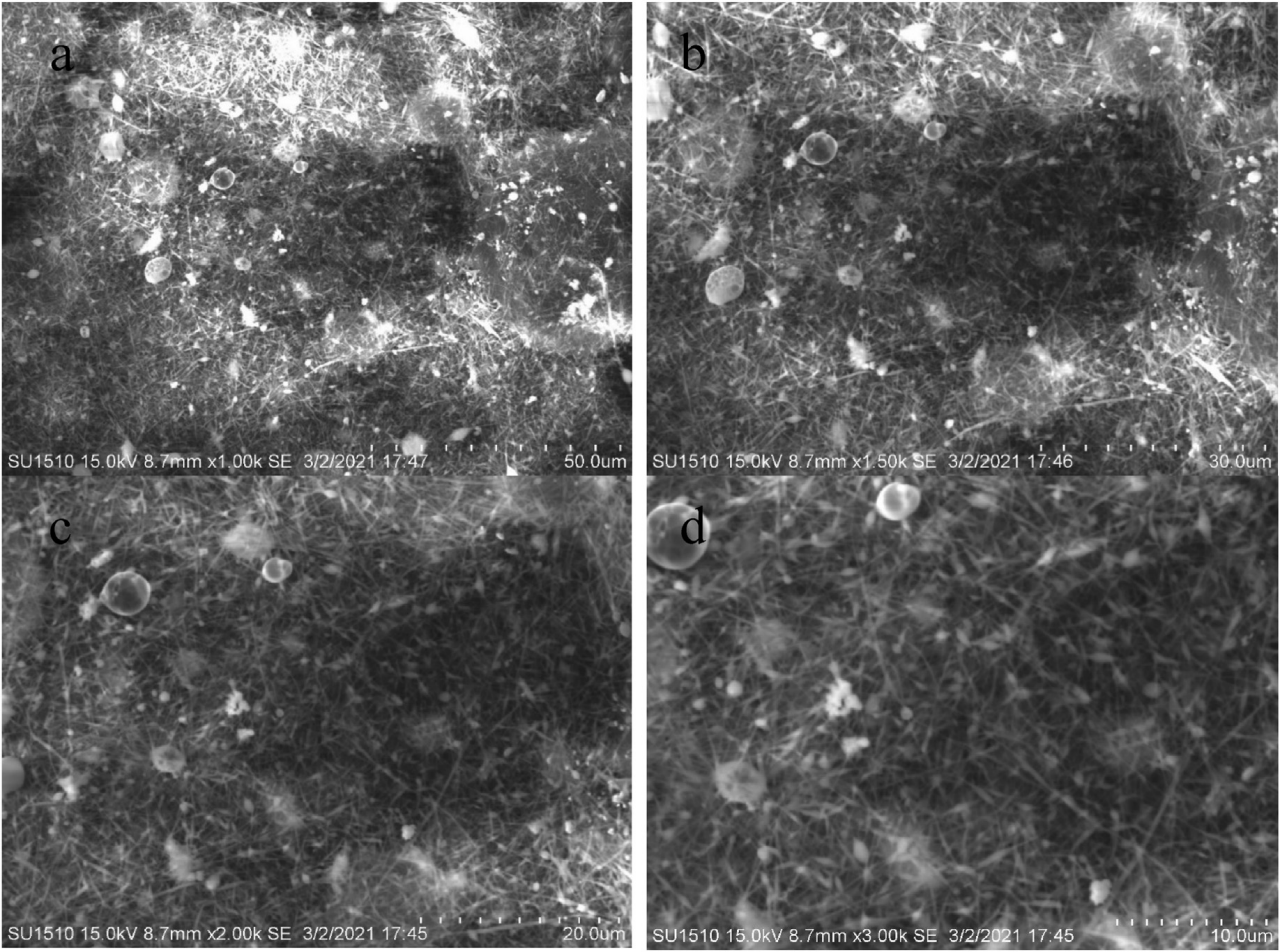
SEM analysis of the developed nanomembrane sample with 15% neem at different resolutions (a) 50 μm, (b) 30 μm, (c) 20 μm, and (d) 10 μm.

**Figure 11:**
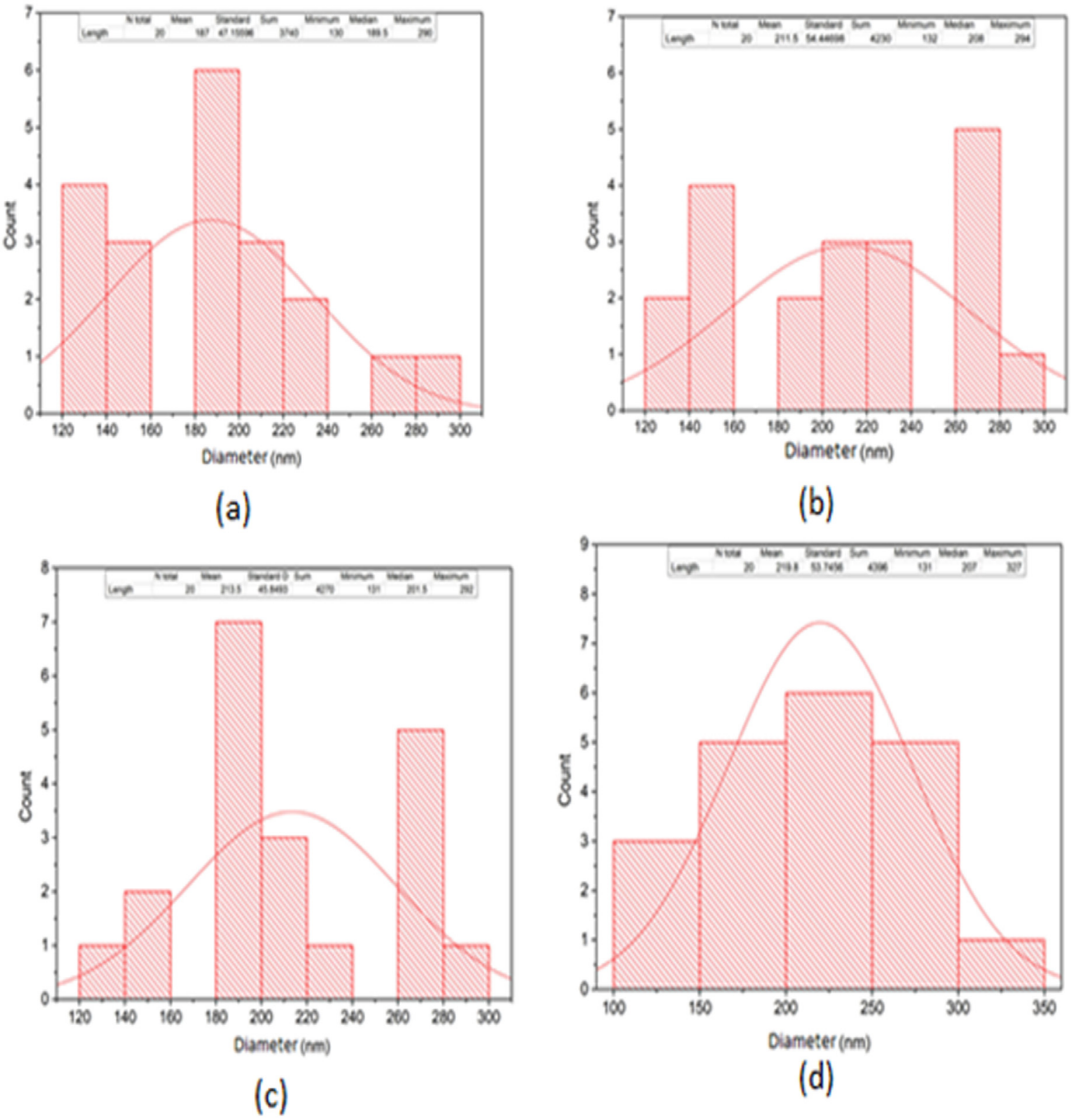
Fiber diameter in fiber-network at (a) 0% Neem, (b) 5% Neem, (c) 10% Neem, (d) 15% Neem.

Figures Figure 7, Figure 8 (a-d), 9 (a-d), and 10 (a-d) show the SEM analysis of the developed nanomembranes with different scales of 50 μm, 30 μm, 20 μm, and 10 μm, and the changes occurred due to the addition of neem at different percentages. The morphology of the electrospun fibers is straight and well proportioned. A smooth fiber surface is observed from the SEM image. However, large gaps among the fibers are seen due to the large diameter among the fibers. The figures show the presence of micropores in the nanomembranes. The formation of micropores in the wound dressing materials is effective due to oxygen diffusion and permeability from air to skin is provided through the micro pores. Air cannot be ventilated through such types of pores and bacteria cannot affect the wound area and thus nullify the surrounding infection effectively [40]. The prepared nanomembranes have uniform, smooth surfaces, and round shape structures [41]. The suitable concentrations as well as condition of the solution were confirmed by the SEM images for uniforms and clear electrospun fibers production that do not contain any microporous nanofibers network or beads [42]. The images also show the presence of dirt and microparticle on the surface. Twenty samples of fibers were considered randomly in different locations of the SEM image and a mean diameter of 187 nm, 211 nm, 213 nm, and 219 nm were collected. It is interesting to observe that with the increase of incorporation of neem extract, the fiber diameters are increased. These results are less or higher than the results of other literature and in almost all cases in nanofibrous membranes the fiber diameters are more than 100 nm [43, 44, 45, 46] (see Figures 9, 10, 11).

### 3.4 Surface topography analysis

**Figure 12:**
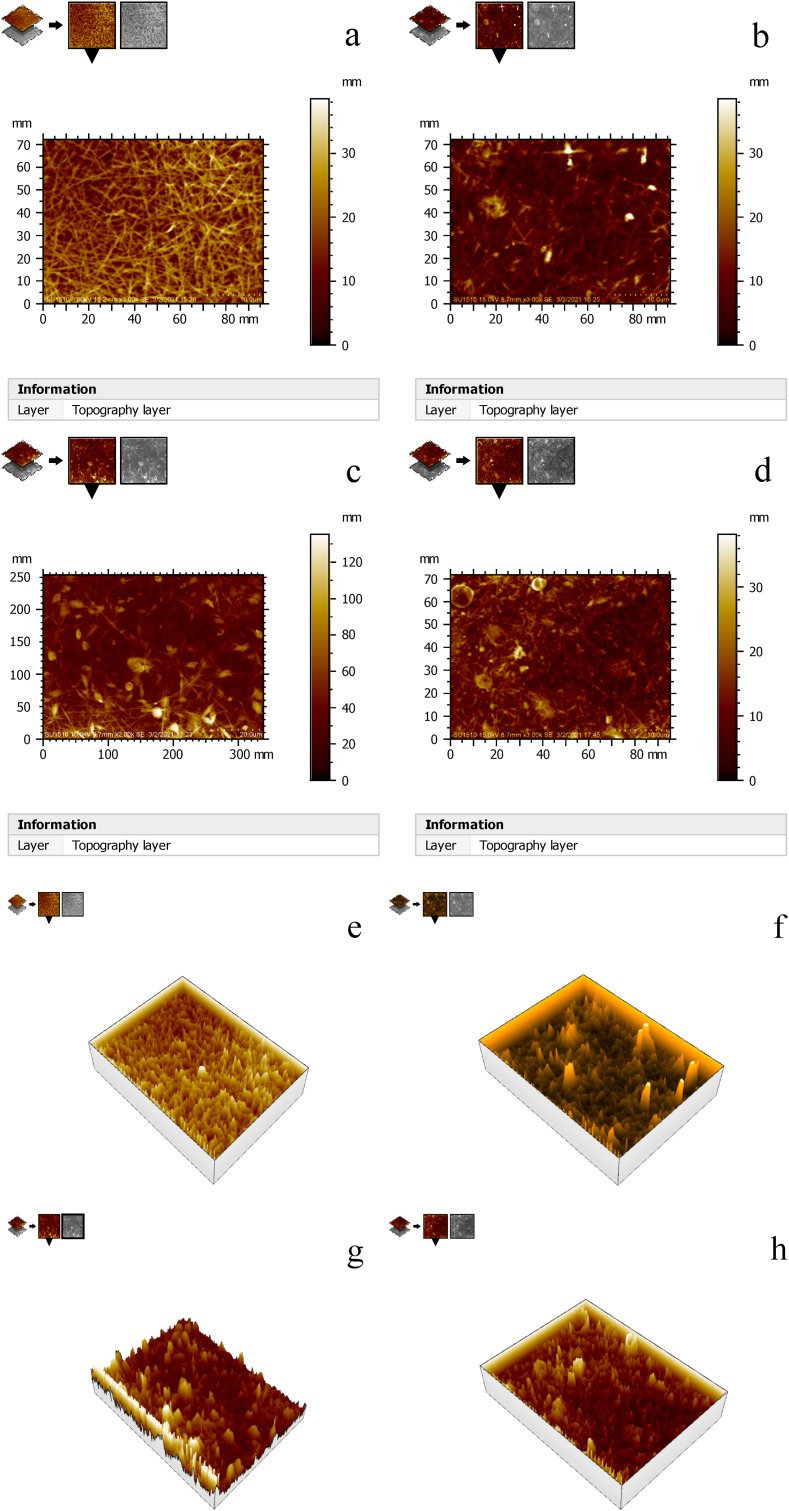
2D surface morphology analysis of the sample with (a) 0% neem, (b) 5% neem, (c) 10% neem and (d) 15% neem and 3D surface morphology analysis of the sample with (e) 0% neem, (f) 5% neem, (g) 10% neem and (h) 15% neem.

Figure 12 (a-h) shows the 2D and 3D surface morphology of the nanomembranes. The 2D morphology images further confirm the formation of micropores in the developed samples. The presence of dirt and micro particle is also confirmed here. The 3D morphology images show the surface roughness by peaks. The darker surface shown by both 2D and 3D surface morphology confirms the presence of neem in the developed nanomembranes. The images also indicate that a lesser number of fibers are observed when neem percentages are increased. The presence of neem is observed with fiber in Figures 12b, 12c, and 12d. Furthermore, more sharp peaks are seen in Figures 12f, 12g, and 12h. The results confirm the sharp peak formation due to the presence of neem in the developed nanomembrane. The images also show that the presence of neem increases the surface of nanomembranes discussed earlier in surface roughness analysis. AFM analysis is important to determine the area of any material that will be used as an implant that will be effective in contact with the biofluid of the dental implant during the bone integration [47].

### 3.5 Particle analysis

**Figure 13:**
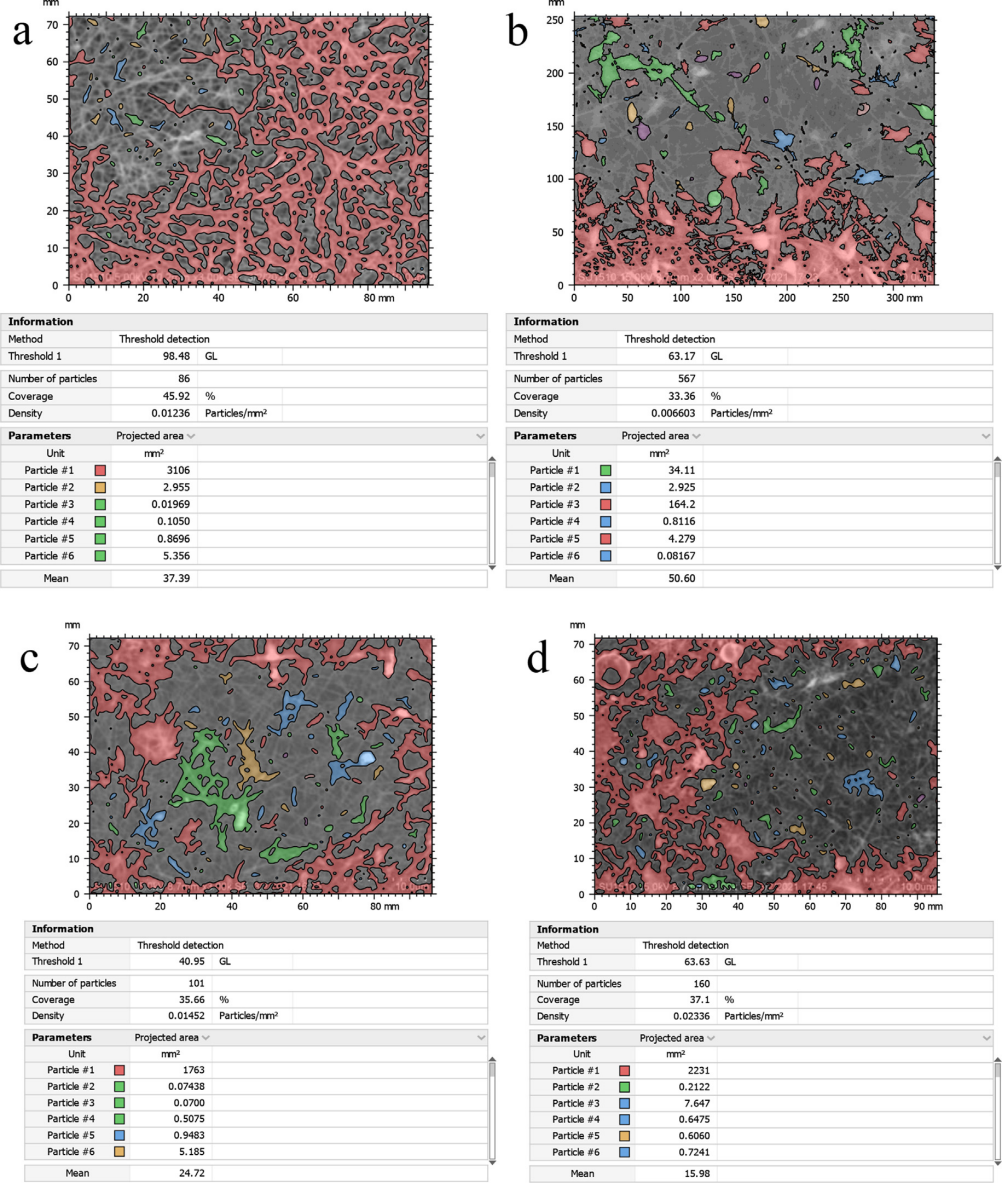
Particle analysis of the nanomembranes with (a) 0% neem, (b) 5% neem, (c) 10% neem and (d) 15% neem.

Partilce analysis observed the presence of PVA, SiC, TiO_2_, Graphene, and neem present on the surface of the nanofibrous membrane shown in SEM images of Figure 13 (a-d). Nanoparticles of different compounds are distributed throughout the surfaces. The absence of neem let the particle be more distributed. However, the presence of neem made the particles congested in some regions and allowed them to be less distributed. Nanomembrane developed by 0% neem shows a lesser number of particles. The results indicate that the addition of neem increased the particle number in the nanomembrane. Besides, density particle/mm^2^ decreased with 5% addition of neem but increased with the addition of 10% and 15% of neem. It is also an indication that the addition of neem increased the density of nanomembrane. However, the mean value increased with 5% addition of neem but decreased with 10% and 15% neem addition.

### 3.6 X-ray diffraction analysis

**Figure 14:**
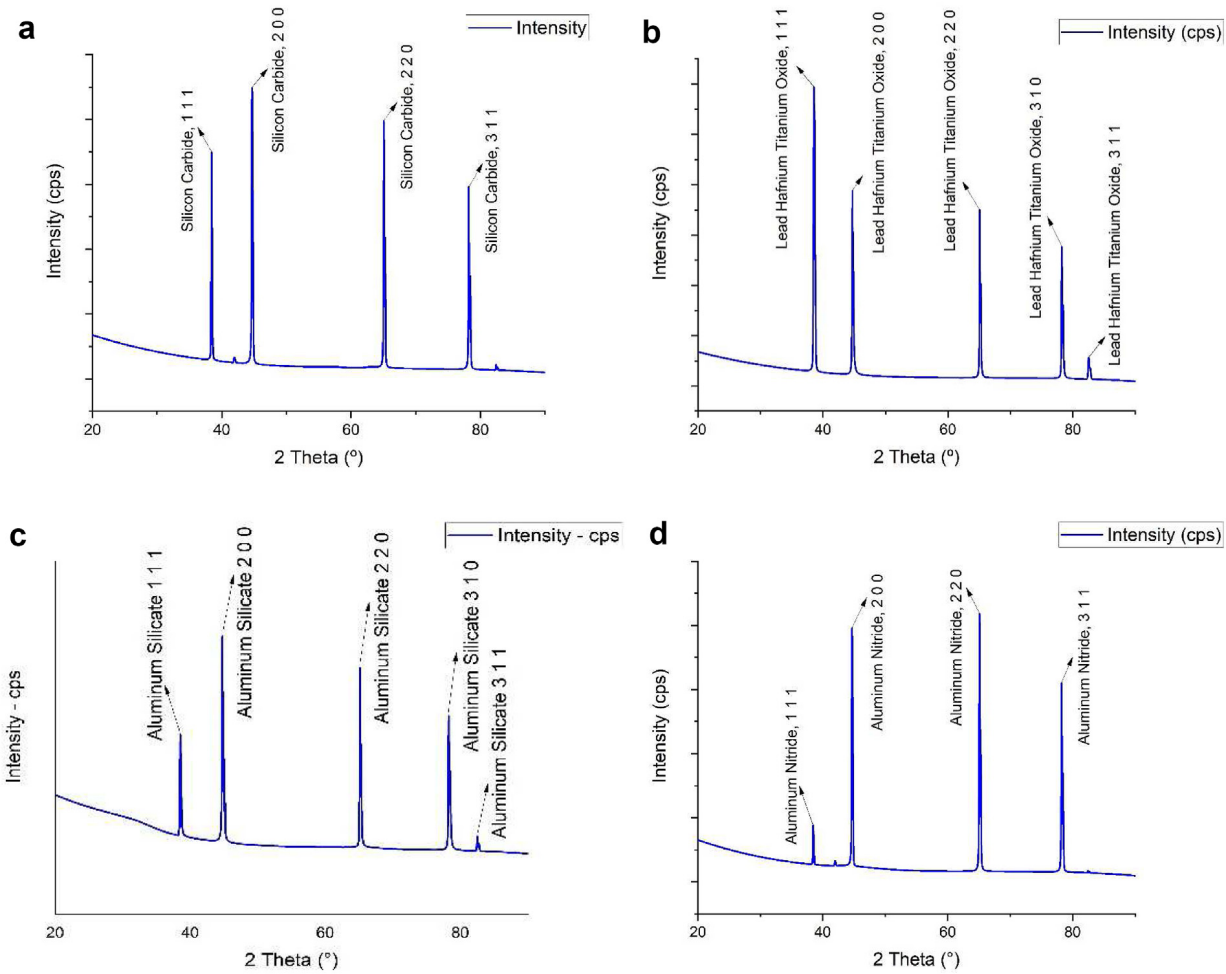
XRD spectra of nanomembrane with (a) 0% Neem, (b) 5% Neem, (c) 10% Neem and (d) 15% Neem.

XRD analysis was performed to determine the crystallinity of the developed nanomembrane films shown in Figure 14 (a-d). Sharp intense peaks are observed in all the figures which confirmed the formation of crystalline structures of the nanomembranes. Atomic position in different crystals is further shown by Miller indices. Broad peaks are observed at around 2θ = 20° which is attributed to the crystalline region attributed by the electrospinning process [48]. Semi-crystalline nature of the nanomembranes were characterized by the aggregates regions of parallel chain segments attributed due to the absence of peaks at around 2θ = 8–10°. The highest relative crystallinity is observed with the samples having 5% and 10% of neem. The sharp peak XRD pattern is almost similar to the other nanofibrous membrane [49, 50].

### 3.7 Antibacterial activity

**Figure 15:**
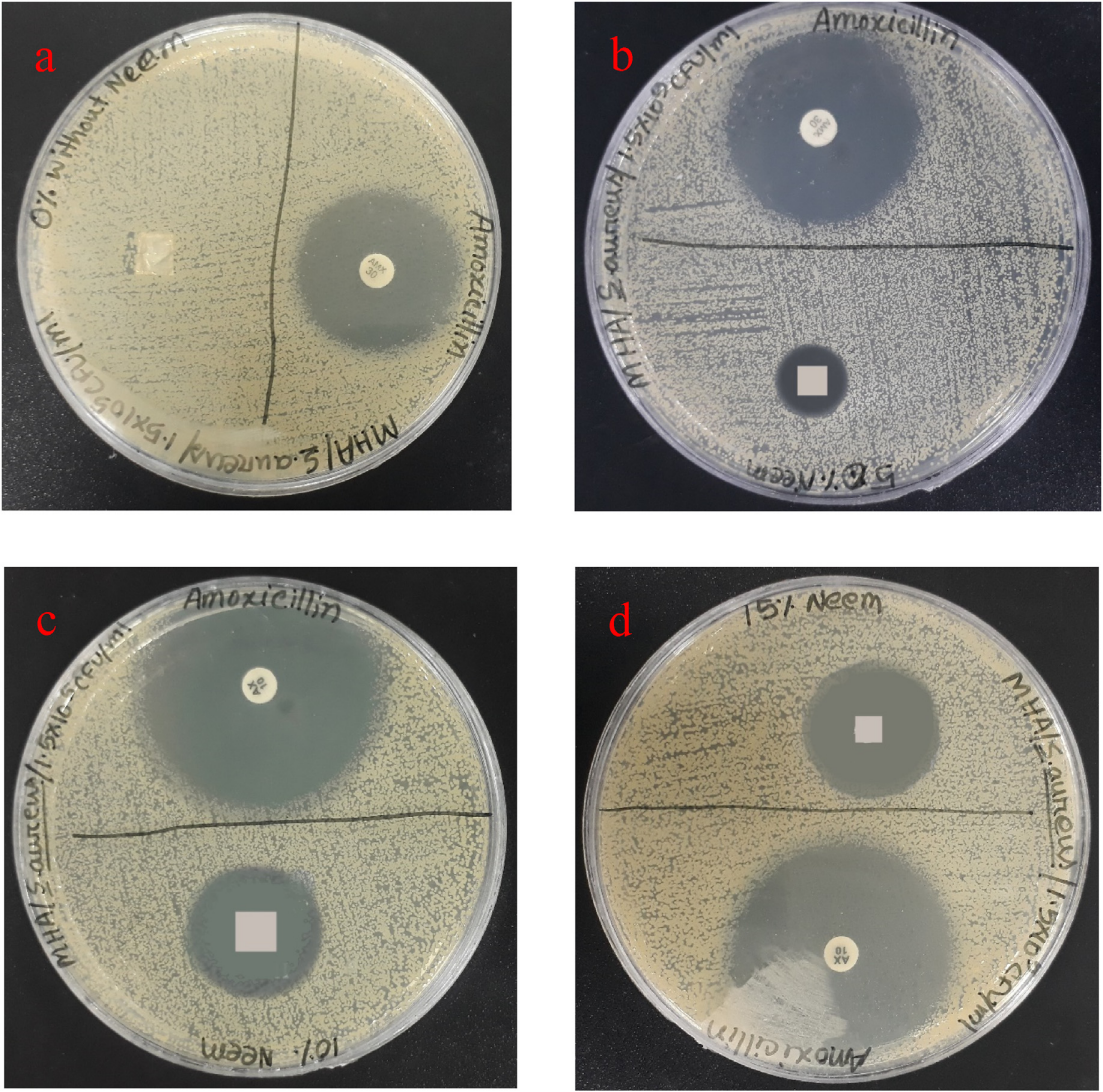
Antibacterial performance of the developed nanomembrane samples at (a) 0% Neem, (b) 5% Neem, (c) 10% Neem, (d) 15% Neem.

The antibacterial activity of the synthesized nanomembrane can be seen in Figure 15 (a-d). The figures confirmed the formation inhibition zone due to the antibacterial activity of the nanomembrane. The formation of a zone without neem in the nanomembrane was not found whereas a zone was found with the nanomembrane having neem as constituents. The increased percentages of neem increased the inhibition zone. The inhibition zone for 0%, 5%, 10%, and 15% neem extracted nanomembranes are 0 mm, 13mm, 16 mm, and 18 mm (from laboratory reports and data) which are higher than the inhibition zone of amoxicillin (mean inhibition zone less than 12 mm) [51]. Normally, the thicker cell wall of *Staphylococcus aureus* makes it resistant and the polycationic group present in the nanomembrane facilitates the disruption of gram-negative bacteria [52, 53]. However, the addition of neem extract enhanced the inhibition zone formation against *Staphylococcus aureus* virus indicating their combined effect. The leaching of antibacterial bioactive constituents from the nanomembrane may cause the incorporation of the neem extract. Besides, bacteria cannot penetrate the mesh structure of the developed nanomembrane due to the presence of micropores and thus the surrounding infections are nullified effectively [54, 55]. The results also indicate that the nanomembrane was capable of inhibiting biofilm formation on all tested strains. However, many studies reported antibacterial properties of neem extract, this study evaluated its effects as a constituent of nanomembrane on *Staphylococcus aureus*. The presence of tetratriterpenoids including azadirachtin in neem made this possible. The inhibition of the *Staphylococcus aureus* production polysaccharide intercellular adhesion is possible by these compounds due to the inhibition of the ica genes expression [56, 57].

### 3.8 Cytotoxicity analysis

The cytotoxicity of the developed nanomembrane was observed on the Vero cell line and the results confirmed the survival of all cell lines. There are no significant changes in the cell line. The study revealed that the developed nanomembrane can safely be used against infections. Ion complexity by legends may be the reason for the zero or near zero toxicity of the developed nanomembrane.

## 4 Conclusion

A new way of developing nanomembrane with the help of electro-spinning techniques has been introduced in this work with antibacterial resistance against *Staphylococcus aureus* with zero or near zero toxic effect. The morphological analysis confirmed the formation of smooth nanofiber with the development of micropores. FTIR spectra demonstrated the characteristic peaks and the peaks became sharper with the increased percentages of neem. Micropores present in the nanomembrane will inhibit the bacteria and keep the skin safe. The addition of neem increased the fiber diameter. Regardless of the percentage of neem, all the developed nanomembranes formed crystal structures confirmed by the XRD analysis. Besides, variation of surface roughness with the various constituent percentages of neem is confirmed by surface roughness analysis. The formation of inhibition zones by the developed nanomembranes was higher than amoxicillin. The overall findings of the present study suggest that the developed nanomembrane can successfully be sued as bio-based antibacterial wound dressing material. This nanofiller-nanofibrous membrane concept can be applied in dental applications with enhanced bacterial, structural, particle distribution, surface, and chemical bonding properties.

## Declarations

### Author contribution statement

Dr. Mohammad Asaduzzaman Chowdhury: Conceived and designed the experiments.

Mr. Nayem Hossain: Performed the experiments; Wrote the paper.

Dr. Md. Abdus Shahid, Md. Jonaidul Alam: Analyzed and interpreted the data.

Sheikh Monir Hossain, Md. Ilias Uddin, Md. Masud Rana: Contributed reagents, materials, analysis tools or data.

### Funding statement

This research did not receive any specific grant from funding agencies in the public, commercial, or not-for-profit sectors.

### Data availability statement

Data will be made available on request.

### Declaration of interests statement

The authors declare no conflict of interest.

### Additional information

No additional information is available for this paper.
